# Polymorphisms in NF-κB pathway genes & their association with risk of lung cancer in the Chinese population

**DOI:** 10.12669/pjms.316.7935

**Published:** 2015

**Authors:** Jing-Wei Zhang, Qiu-Sheng Chen, Jian-Xia Zhai, Peng-Ju Lv, Xiao-Yan Sun

**Affiliations:** 1 Jing-Wei Zhang, MD. Department of Respiratory, Zhengzhou Central Hospital Affiliated to Zhengzhou University, Zhengzhou 450007, China; 2 Qiu-Sheng Chen, MD. Department of Respiratory, Zhengzhou Central Hospital Affiliated to Zhengzhou University, Zhengzhou 450007, China; 3 Jian-Xia Zhai, MD. Department of Respiratory, Zhengzhou Central Hospital Affiliated to Zhengzhou University, Zhengzhou 450007, China; 4 Peng-Ju Lv, MD. Translational Medicine Center, Zhengzhou Central Hospital Affiliated to Zhengzhou University, Zhengzhou 450007, China; 5 Xiao-Yan Sun, MD. Translational Medicine Center, Zhengzhou Central Hospital Affiliated to Zhengzhou University, Zhengzhou 450007, China

**Keywords:** Genetic variation, Lung cancer, NF-κB, Polymorphism, Risk

## Abstract

**Objective:**

To investigate the association of *NFKB1* -94 ins/del ATTG, *NFKBIA* -826C>T and *NFKBIA* -881A>G polymorphisms with risk of lung cancer in a Chinese population.

**Methods:**

Genotyping of the polymorphisms were performed on 1,436 subjects (718 cases and 718 controls) by using PCR-RFLP technique, followed by DNA sequencing.

**Results:**

We found a significant risk reduction associated with heterozygous ins/del (OR=0.705, 95% CI=0.566-0.878, P=0.002) and variant del/del (OR=0.342, 95% CI=0.221-0.528, P<0.001) genotypes of the *NFKB1* polymorphism. In contrast, the heterozygous and variantgenotypes of the*NFKBIA* polymorphisms showed association with increased lung cancer risk (*NFKBIA* -826 CT,OR=1.256, 95%CI=1.004-1.572, P=0.046; TT,OR=1.773, 95% CI=1.131-2.778, P=0.013; *NFKBIA* -881 AG,OR=1.277, 95% CI=1.023-1.599, P=0.031; GG,OR=1.801, 95% CI=1.169-2.775, P=0.008). Several genotypic combinations of the three polymorphisms also showed significant association with lung cancer risk. The risk association of *NFKB1* polymorphism remained significant when analyses were done according to gender and smoking status (P<0.05). The significance of *NFKBIA* risk association was not observed when gender-specific analyses were made (P>0.05), while only *NFKBIA* -881 GG genotype showed significant risk association among smokers when analyzed according to smoking status (P=0.032).

**Conclusions:**

Polymorphisms in *NFKB1* and *NFKBIA*genes were associated with risk of lung cancer.

## INTRODUCTION

Lung cancer is the most common type of cancer worldwide and represents a leading cause of cancer-related mortality.^1^-^3^ It has been suggested that genetic variations of cancer-related genes could play a role in influencing individual susceptibility to lung cancer.^4^,^5^ Among the many candidate genes, polymorphisms within inflammatory responsegenes have received increasing attention in the past few years, since inflammation has been strongly implicated in carcinogenesis.^6^-^8^

The nuclear factor-kappa B (NF-κB) family constitutes a group of transcription factors which serve as important mediators in inflammatory responses. In addition to inflammatory responses, a wide range of signal transduction processes converge on the NF-κB pathway, including cell proliferation, apoptosis, angiogenesis and many others.^9^ The p105/p50 isoforms of the NF-κBfamily, denoted NF-κB1, represents the most ubiquitous form of transcription factor in the family. Given the important role of NF-κB1 as the central hub of many biological processes, it is closely regulated by its endogenous inhibitors IκBα under normal conditions.^10^ NF-κB1 and IκBα are encoded by the *NFKB1* and *NFKBIA* genes respectively, and it has been shown that disrupted expressions of these genes can result in carcinogenesis.^11^-^13^

Functional polymorphisms within the promoter region of these genes could potentially influence the levels of the proteins encoded. These functional polymorphisms may therefore contribute to the inter individual differences in lung cancer risk. To test our hypothesis, we conducted a case-control study to investigate the association of *NFKB1* -94 ins/del ATTG polymorphism and *NFKBIA* -826C>T and -881A>G polymorphisms with the risk of lung cancer in a Chinese population.

## METHODS

### Study Population

718 lung cancer patients and 718controls participated in this retrospective case-control study. The participants were recruited between September 2011 andAugust 2014 fromthe Zhengzhou Central Hospital Affiliated to Zhengzhou University. Controls were cancer-free individuals randomly selected from a cancer screening program, and were matched (in frequency) to cases by age, sex and smoking behavior. The study was approved by the Research Review and Ethics Board of Zhengzhou Central Hospital Affiliated to Zhengzhou University(Approval number: 21234/RESP/43.2011). Written informed consent was obtained from all the participants prior to the study.

### Genotyping

**Fig.1:**
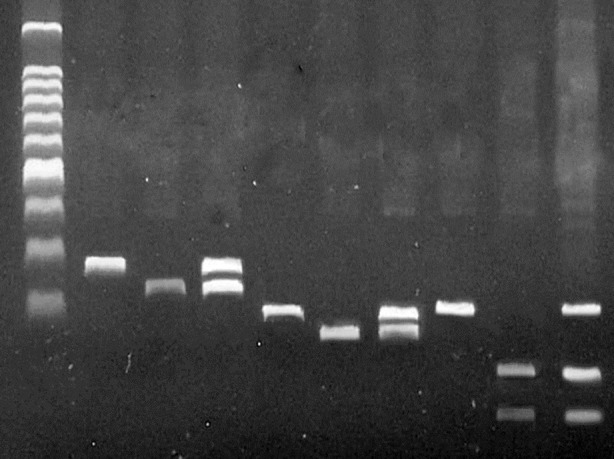
Gel image of PCR-RFLP. From left to right, NFKB1 del/del, NFKB1 ins/ins, NFKB1 ins/del, NFKBIA -826TT, NFKBIA -826CC, NFKBIA -826CT, NFKBIA -881AA, NFKBIA -881GG, NFKBIA -881AG.

Genotyping was performed by using the polymerase chain reaction-restriction fragment length polymorphism (PCR-RFLP) method (Fig.1)described elsewhere using the Life Express thermocycler (Bioer, China), and the genotypes were confirmed by sequencing 10% of the PCR products.^14^,^15^

### Statistical Analysis

Differences in age, smoking status and gender between cases and controls were evaluated using the chi-square test. The association between the polymorphisms and lung cancer risk was determined using the logistic regression method to assess the odds ratio (OR). P values less than 0.05 were considered statistically significant.

## RESULTS

### Association of NFKB1 and NFKBIA polymorphisms with lung cancer risk

The association between *NFKB1* and *NFKBIA* polymorphisms and lung cancer risk is shown in Table-I. By using ins/ins genotype as the reference, the heterozygous and homozygous variant genotypes of the *NFKB1* polymorphism were found to be significantly associated with the reduced risk of lung cancer(P=0.002 for heterozygous, P<0.001 for variant). On the other hand, the heterozygous and homozygous variant genotypes of the *NFKBIA* polymorphisms appeared to be significantly associated with increased lung cancer risk(P=0.046 for -826CT, P=0.013 for -826TT, P=0.031 for -881AG, P=0.008 for -881GG,).

**Table-I:** Association between NFKB1 -94 ins/del ATTG polymorphism, NFKBIA -826C>Tpolymorphism and NFKBIA -881A>G polymorphism and lung cancer risk.

Genotype	Case, N=718	Control, N=718	OR (95% CI)	P
-94 ins/del ATTG				
Ins/ins	434(60.4%)	352(49.0%)	1.000	-
Ins/del	252(35.1%)	290(40.4%)	0.705 (0.566-0.878)	0.002*
Del/del	32(4.5%)	76(10.6%)	0.342 (0.221-0.528)	<0.001*
-826C>T				
CC	413(57.5%)	461(64.2%)	1.000	-
CT	251(35.0%)	223(31.1%)	1.256 (1.004-1.572)	0.046*
TT	54(7.5%)	34(4.7%)	1.773 (1.131-2.778)	0.013*
-881A>G				
AA	402(56.0%)	454(63.2%)	1.000	-
AG	257(35.8%)	227(31.6%)	1.277 (1.023-1.599)	0.031*
GG	59(8.2%)	37(5.2%)	1.801 (1.169-2.775)	0.008*

* significant.

### Combinations of polymorphisms and lung cancer risk

The association of combinations of *NFKB1* -94 ins/del ATTG polymorphism and *NFKBIA* -826C>T polymorphism with lung cancer risk is shown in Table-II. Of the nine possible combinations, significant risk association was observed only for ins/del-CC(P=0.006), del/del-CC(P<0.001), and del/del-CT (P=0.003)combination genotypes. All the three combination genotypes showed decreased risk association.

**Table-II:** Association between combinations of NFKB1 -94 ins/del ATTG polymorphism and NFKBIA -826C>T polymorphism and lung cancer risk.

Genotype		Case	Control	OR (95% CI)	P
-94 ins/del ATTG	-826C>T				
Ins/ins	CC	259 (36.1%)	232 (32.3%)	1.000	--
Ins/del	CC	134 (18.7%)	179 (24.9%)	0.671 (0.504-0.892)	0.006*
Del/del	CC	20 (2.8%)	50 (7.0%)	0.358 (0.207-0.620)	<0.001*
Ins/ins	CT	144 (20.1%)	103 (14.3%)	1.252 (0.920-1.705)	0.153
Ins/del	CT	99 (13.8%)	95 (13.2%)	0.934 (0.669-1.302)	0.685
Del/del	CT	8 (1.1%)	25 (3.5%)	0.287 (0.127-0.648)	0.003*
Ins/ins	TT	31 (4.3%)	17 (2.4%)	1.633 (0.881-3.029)	0.119
Ins/del	TT	19 (2.6%)	16 (2.2%)	1.064 (0.535-2.117)	0.860
Del/del	TT	4 (0.6%)	1 (0.1%)	3.583 (0.398-32.288)	0.255

* significant.

Association between combinations of *NFKB1* -94 ins/del ATTG polymorphism and *NFKBIA* -881A>G polymorphism and lung cancer risk is shown in Table-III. Similar to the above, three combinations showed significantly decreased risk association with lung cancer, namely ins/del-AA(P=0.004), del/del-AA (P<0.001)and del/del-AG (P=0.001)genotypes.

**Table-III:** Association between combinations of NFKB1 -94 ins/del ATTG polymorphism and NFKBIA -881A>G polymorphism and lung cancer risk.

Genotype		Case	Control	OR (95% CI)	P
-94 ins/del ATTG	-881A>G				
Ins/ins	AA	254 (35.4%)	228 (31.8%)	1.000	--
Ins/del	AA	128 (17.8%)	176 (24.5%)	0.653 (0.489-0.872)	0.004*
Del/del	AA	20 (2.8%)	50 (7.0%)	0.359 (0.208-0.621)	<0.001*
Ins/ins	AG	148 (20.6%)	105 (14.6%)	1.265 (0.930-1.721)	0.134
Ins/del	AG	103 (14.3%)	98 (13.6%)	0.943 (0.679-1.311)	0.729
Del/del	AG	6 (0.8%)	24 (3.3%)	0.224(0.090-0.559)	0.001*
Ins/ins	GG	32(4.5%)	19 (2.6%)	1.512(0.834-2.741)	0.174
Ins/del	GG	21 (2.9%)	16 (2.2%)	1.178 (0.600-2.313)	0.634
Del/del	GG	6 (0.8%)	2 (0.3%)	2.693(0.538-13.476)	0.228

* significant.

The combinations of *NFKBIA* -826C>T polymorphism and *NFKBIA* -881A>G polymorphism and their association with lung cancer risk is shown in Table-IV. Of the nine possible combinations, two combinations showed significantly increased risk association, namely CT-AG (P=0.043)and TT-GG (P=0.017)genotypes.

**Table-IV:** Association between combinations of NFKBIA -826C>T polymorphism and NFKBIA -881A>G polymorphism and lung cancer risk.

Genotype		Case	Control	OR (95% CI)	P
-826C>T	-881A>G				
CC	AA	400 (55.7%)	452 (63.0%)	1.000	--
CT	AA	2 (0.3%)	2 (0.3%)	1.130(0.158-8.059)	0.903
TT	AA	0 (0.0%)	0 (0.0%)	---	--
CC	AG	8 (1.1%)	6 (0.8%)	1.507(0.518-4.380)	0.452
CT	AG	246 (34.3%)	220 (30.6%)	1.264(1.008-1.584)	0.043*
TT	AG	3 (0.4%)	1 (0.1%)	3.390 (0.351-32.722)	0.291
CC	GG	5 (0.7%)	3 (0.4%)	1.883(0.447-7.931)	0.388
CT	GG	3 (0.4%)	1 (0.1%)	3.390 (0.351-32.722)	0.291
TT	GG	51 (7.1%)	33 (4.6%)	1.746(1.105-2.761)	0.017*

* significant.

### Analysis of NFKB1 risk association by gender and smoking status

The stratified association between the *NFKB1* polymorphism and lung cancer risk according to gender and smoking status is shown in Table-V. The heterozygote genotype appeared to decrease the risk of lung cancer among females(P=0.005), but not males. However, the variant genotype decreased the lung cancer risk both in males(P=0.004) and females(P<0.001). The heterozygous genotype was also associated witha decreased risk of lung cancer among smokers(P=0.003), but not non-smokers. The variant genotype was associated with decreased lung cancer risk in both smokers (P<0.001)and non-smokers(P=0.001).

**Table-V:** Association between NFKB1 -94 ins/del ATTG polymorphism and lung cancer risk according to sex and smoking status.

Genotype	Case	Control	OR (95% CI)	P
Male				
Ins/ins	220 (58.8%)	188 (50.3%)	1.000	--
Ins/del	137 (36.6%)	150 (40.1%)	0.781 (0.577-1.056)	0.108
Del/del	17 (4.5%)	36 (9.6%)	0.404 (0.220-0.742)	0.004*
Female				
Ins/ins	214 (62.2%)	164 (47.7%)	1.000	--
Ins/del	115 (33.4%)	140 (40.7%)	0.630 (0.457-0.867)	0.005*
Del/del	15 (4.4%)	40 (11.6%)	0.287 (0.154-0.538)	<0.001*
Smoker				
Ins/ins	234 (60.2%)	181 (46.5%)	1.000	--
Ins/del	135 (34.7%)	164 (42.2%)	0.637 (0.472-0.859)	0.003*
Del/del	20 (5.1%)	44 (11.3%)	0.352 (0.200-0.617)	<0.001*
Non-smoker				
Ins/ins	200 (60.8%)	171 (52.0%)	1.000	---
Ins/del	117 (35.6%)	126 (38.3%)	0.794 (0.574-1.098)	0.163
Del/del	12 (3.6%)	32 (9.7%)	0.321 (0.160-0.642)	0.001*

* significant.

### Analysis of NFKBIA -826C>T risk association by gender and smoking status

The association between *NFKBIA* -826C>T polymorphism and lung cancer risk according to sex and smoking status is shown in Table-VI.. Interestingly, the risk association of the heterozygous and variant genotypes appeared to be lost after stratification by gender and smoking status.

**Table-VI:** Association between NFKBIA -826C>T polymorphism and lung cancer risk according to sex and smoking status.

Genotype	Case	Control	OR (95% CI)	P
Male				
CC	215 (57.5%)	241 (64.4%)	1.000	---
CT	133 (35.6%)	116 (31.0%)	1.285 (0.943-1.752)	0.112
TT	26 (7.0%)	17 (4.5%)	1.714 (0.905-3.246)	0.098
Female				
CC	198 (57.6%)	220 (64.0%)	1.000	---
CT	118 (34.3%)	107 (31.1%)	1.225 (0.886-1.695)	0.220
TT	28 (8.1%)	17 (4.9%)	1.830 (0.972-3.445)	0.061
Smoker				
CC	219 (56.3%)	245 (63.0%)	1.000	---
CT	138 (35.5%)	124 (31.9%)	1.245 (0.919-1.686)	0.157
TT	32 (8.2%)	20 (5.1%)	1.790 (0.995-3.222)	0.052
Non-smoker				
CC	194 (59.0%)	216 (65.7%)	1.000	---
CT	113 (34.3%)	99 (30.1%)	1.271 (0.912-1.772)	0.157
TT	22 (6.7%)	14 (4.3%)	1.750 (0.871-3.515)	0.116

* significant.

### Analysis of NFKBIA -881A>G risk association by gender and smoking status

The association between *NFKBIA* -881A>G polymorphism and the risk of lung cancer according to sex and smoking status is shown in Table-VII. Similar to the *NFKBIA* -826C>T polymorphism, no association was observed for the heterozygous and variant genotypes and lung cancer risk in both males and females after stratification by sex. However, after stratification by smoking status, significant association was observed only for the variant genotype with lung cancer risk among smokers(P=0.032).

**Table-VII:** Association between NFKBIA -881A>G polymorphism and lung cancer risk according to sex and smoking status.

Genotype	Case	Control	OR (95% CI)	P
Male			
AA	208 (55.6%)	235 (62.8%)	1.000	---
AG	136 (36.4%)	120 (32.1%)	1.280 (0.941-1.743)	0.116
GG	30 (8.0%)	19 (5.1%)	1.784 (0.975-3.264)	0.060
Female				
AA	194 (56.4%)	219 (63.7%)	1.000	---
AG	121 (35.2%)	107 (31.1%)	1.277 (0.923-1.765)	0.140
GG	29 (8.4%)	18 (5.2%)	1.819 (0.979-3.378)	0.058
Smoker				
AA	214 (55.0%)	240 (61.7%)	1.000	---
AG	140 (36.0%)	128 (32.9%)	1.227 (0.907-1.660)	0.185
GG	35 (9.0%)	21 (5.4%)	1.869 (1.055-3.310)	0.032*
Non-smoker				
AA	188 (57.1%)	214 (65.0%)	1.000	---
AG	117 (35.6%)	99 (30.1%)	1.345 (0.966-1.874)	0.080
GG	24 (7.3%)	16 (4.9%)	1.707 (0.881-3.311)	0.113

* significant.

## DISCUSSION

Given the important role of NF-κB and IκB in diverse biological pathways, genetic variations within key genes of the NF-κB pathway could obstruct the normal functioning of the protein products which in turn influences the risk of cancers. The *NFKB1* -94 ins/del ATTG, *NFKBIA* -826C>T and *NFKBIA* -881A>G polymorphisms have been described. These functional polymorphisms have been shown to affect the level of the proteins produced, with the deletion allele, T allele and G allele of the three polymorphisms are associated with the reduced production of the respective proteins products.^16^,^17^

We have showed that the variant del genotype of the *NFKB1* -94 ins/del ATTG polymorphism could significantly reduce lung cancerrisk. Our results are in disagreement with those reported by Huang et al.^18^ who found no association between the *NFKB1* polymorphism and lung cancer risk in a Chinese population. However, our results concur with Huo et al.^19^ who investigated the association of the polymorphism with ovarian cancer among Chinese.

We have also showed in the current study that the variant genotypes of *NFKBIA* -826C>T and -881A>G polymorphisms could increase lung cancerrisk. Similar to the *NFKB1* -94 ins/del ATTG polymorphism, our results are contrary to the results of Huang et al.^18^ who also found no association between the -826C>T polymorphism and lung cancer risk. However, when stratified by gender, we found that our results concur with the former, in that there was a lack of association of the polymorphism and cancer risk in both males and females. A similar lack of association was observed when stratified by smoking status, with the exception of *NFKBIA* -881GG variant genotype among smokers. This was in partial agreement with Umar et al.^20^ who found that the CT and CT+TT genotype of the -826C>T polymorphism was associated with a decreased risk of esophageal squamous cell carcinoma in an Indian population. On the other hand, Lin et al.^21^ showed that the heterozygous genotype of both the *NFKBIA* -826C>T and -881A>G polymorphisms increased the risk of oral cancer among Chinese. The disagreements among the findings reported in the literature could be due to the different backgrounds (age, sex, ethnic group, smoking status and geography) of the study subjects recruited, as well as the properness of subject matching.

The strengths of this study are: (i) it is the first study which establishes an association between the polymorphism and lung cancer risk in China, and (ii) it was conducted in a relatively properly-matched population. However, the limitation is that only three SNPs were analyzed.

## CONCLUSION

We have showed that the variant alleles of *NFKB1* -94 ins/del ATTG polymorphism, *NFKBIA* -826C>T polymorphism and *NFKBIA* -881A>G polymorphism could influence the risk of lung cancer in China.
